# Eph Receptors and Ephrin Ligands: Important Players in Angiogenesis and Tumor Angiogenesis

**DOI:** 10.1155/2010/135285

**Published:** 2010-03-10

**Authors:** Birgit Mosch, Bettina Reissenweber, Christin Neuber, Jens Pietzsch

**Affiliations:** Department of Radiopharmaceutical Biology, Institute of Radiopharmacy, Research Center Dresden-Rossendorf, 01328 Dresden, Germany

## Abstract

Eph receptors and their ephrin ligands were identified in the late 1980's. Subsequently, they were linked to different physiological and pathophysiological processes like embryonic development, angiogenesis, and tumorigenesis. In this regard, recent work focused on the distribution and effects of Eph receptors and ephrins on tumor cells and tumor microenvironment. The purpose of this review is to outline the role of these molecules in physiological angiogenesis and pathophysiological tumor angiogenesis. Furthermore, novel therapeutical approaches are discussed as Eph receptors and ephrins represent attractive targets for antiangiogenic therapy.

## 1. Eph Receptors and Ephrins

### 1.1. Structure and Signaling

**Figure 1:**
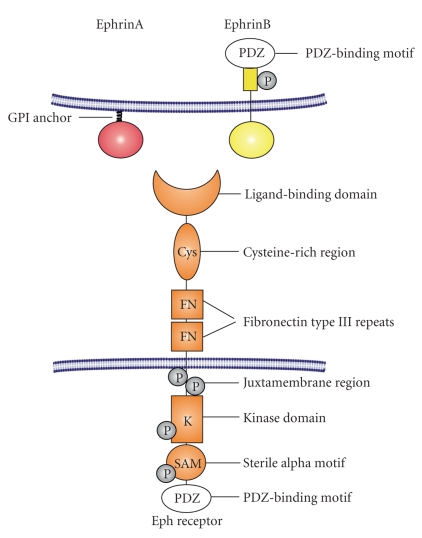
Structure of Eph receptors and ephrin ligands. PDZ: Postsynaptic density 95-Discs large-Zonula occludentes-1-protein, GPI: glycosylphosphatidylinositol.

**Figure 2:**
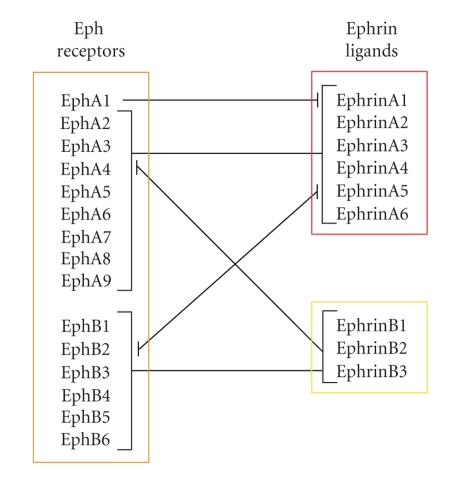
Major interactions of Eph receptors and ephrin ligands.

Eph receptors were identified in the late 1980's [1] and are known as largest family of receptor tyrosine kinases. They consist of a glycosylated extracellular domain with the immunoglobulin-like ligand-binding site, followed by a cysteine-rich region and two fibronectin type III repeats (Figure 1). Connected via a single transmembrane spanning domain, the intracellular region contains a juxtamembrane domain, a tyrosine kinase domain, a sterile alpha motif, and a PDZ-(Postsynaptic density 95-Discs large-Zonula occludentes-1) binding motif [1, 2]. Eph receptors bind membrane bound ligands, the ephrins, and both, receptors and ligands, are divided into two subclasses A or B based on binding properties and structural homologies. Class A ephrins are membrane-bound via a glycosylphosphatidylinositol anchor and class B ephrins contain a transmembrane domain and a short cytoplasmic region with conserved tyrosine residues and a PDZ-binding motif. Class A Eph receptors preferentially bind all A-type ephrins and class B Eph receptors bind all B-type ligands. However, there are some exceptions, as EphA1 primarily binds ephrinA1, EphA4 binds both, A- and B-type ligands, and ephrinA5 binds EphA receptors as well as EphB2 (Figure 2) [3–6]. Until today, 15 different receptors and 9 ligands are known (https://eph-nomenclature.med.harvard.edu/table_1.html). 

In contrast to other receptor tyrosine kinases, Eph receptors/ephrins show unique properties in their activation and signaling. For the activation of the receptors not only dimerization as in most receptor tyrosine kinases is required but also multimerization of the ligands [7]. Multimer-induced signaling seems to be different from signals of normal dimers in so far as the degree of multimerization of the ephrins accounts for the kind and strength of biological effects [8]. As Eph receptors bind ligands which are also membrane bound, cell-cell contact is needed for Eph receptor activation. On the other hand, recent work demonstrated that at least A-type ephrins can be released from the cell surface [9, 10]. These soluble proteins were shown to be functionally active and possibly represent an additional signaling mechanism without mandatory cell contact. Nevertheless, Eph receptor/ephrin signaling can also proceed bidirectionally, “forward” and “reverse” [11–13]. “Forward signaling” involves binding of ephrins by the appropriate Eph receptor. This leads to autophosphorylation of intracellular tyrosine residues of the Eph receptor and further to activation of different downstream signal transduction cascades [14, 15]. In the case of B-type ephrins, signaling can also take place “reverse”, if the cytoplasmic tail of the ephrin is phosphorylated which also results in activation of different signaling cascades. Moreover, it should be noted that ephrinA ligands might also have the potency to reverse signaling (overview in [4]). Many studies of the last decade indicate a complex cross-talk between Eph receptors/ephrins and other signaling pathways which is necessary for consistent biological functions. The interactions between Eph receptors/ephrins and different cell surface receptors, adhesion molecules, channels, pores, and cell surface proteases are reviewed in [16].

Taken together, Eph receptors and their appropriate ephrin ligands represent an essential communication system that directs cell motility, repulsion and adhesion, cell-cell and cell-matrix contacts in a number of biological processes. Due to the focused topic of this article, only two of them, angiogenesis and tumor angiogenesis, should be elucidated in detail, while other processes will be outlined in brief.

### 1.2. Embryonic and Neural Development

Eph receptor/ephrin signaling plays a crucial role in embryonic development [17]. As an example, it has been shown that altered expression of EphA3 and ephrinA5 leads to defects in gastrulation and somite development [18]. Furthermore, together with integrin-*α*5 and fibronectin, Eph receptors/ephrins are discussed to mediate mesenchymal-to-epithelial transition and, hence, formation of somite boundaries [19]. 

A further role of Eph receptor/ephrin signaling is suggested in the developing and adult vertebrate brain. Due to their complementary expression pattern, EphA4, EphB2, EphB3, and their B-type ligands are implicated in the formation of rhombomere boundaries. Thereby, bidirectional signaling seems to be required for the restriction of cell intermingling between neighboring rhombomeres [20, 21]. Furthermore, many studies analyzed the involvement of Eph receptor/ephrin signaling in neuronal growth cone collapse, leading to axon guidance by inhibition [17]. For instance, studies on EphA4- and ephrinB3-null mice indicated that both proteins are required for normal formation of the corticospinal tract fibres, whereby Eph receptor forward signaling is mandatory [22, 23]. The importance of proper ephrin ligand expression for correct outgrowth of retinal ganglion cell axons was analyzed by Hornberger and colleagues. They demonstrated that unscheduled overexpression of ephrinA2 in temporal axons leads to insensitivity of guiding outgrowing axons of the caudate tectum by repulsion [24]. In the development of the visual system it was shown that EphB2 and EphB3 receptors and B-type ephrins are involved in axon pathfinding of retinal ganglion cells to the optic disc and that deletion of both EphB2 and EphB3 leads to increased frequency of axon guidance errors in this model [25]. Furthermore, the EphB2 receptor is also involved in synaptic functions (synaptic plasticity) in the adult mammalian central nervous system [26, 27]. In this regard, Henderson and colleagues found that mice lacking the EphB2 receptor show reduced synaptic *N*-methyl-*D*-aspartate-mediated current and reduced long-term potentiation in hippocampal and dentate gyrus synapses [28].

### 1.3. Tumorigenesis

Eph receptor/ephrin signaling plays not only a role in physiological processes, but also in pathophysiological processes such as tumorigenesis [7, 29]. Thus, many ephrins and Eph receptors were found to be upregulated in several human carcinomas such as breast, colon, liver, prostate, and melanoma and are often associated with tumor progression and metastasis (for overview see [7, 29–31]). On the other hand, also downregulation of Eph receptors can lead to increased metastasis and carcinogenesis as shown for EphA1 in colorectal cancer, EphA7 in prostate carcinomas, and EphB6 in melanoma [32–34]. Thereby, Eph receptors do not operate like classical oncogenic growth factor receptors, because their activation does only in exceptional cases influence proliferation of the tumor cells [35, 36]. Rather dysregulation of Eph receptor activity seems to effect cell-matrix attachment, cell-cell attachment, organization of the cytoskeleton, and modification of tumor cell survival, which could result in increased cellular motility, tumor cell invasion, and metastasis. Cell-matrix attachment can be influenced by Eph receptors via modulating the integrin activity. For instance, EphA2 stimulation with ephrinA1 leads to decreased focal adhesion kinase (FAK) phosphorylation which further results in inactive conformation of integrins and, finally, inhibition of integrin-mediated adhesion, cell spreading, and migration [37]. It is assumed that also small GTPases of the Ras and Rho family could be linked to decreased integrin activation and cellular adhesion [38]. However, the modification of cell attachment is probably dependent on the Eph receptor/ephrin ligand ratio. A high expression of Eph receptor and low expression of ephrin ligand could represent an “advantage” for tumor growth and metastasis [29]. A possible cause for imbalanced Eph receptor/ephrin ratio was recently analyzed by Winter and colleagues who identified binding sites of multiple mRNA-stabilizing and destabilizing factors at the 3'UTR sequences of Eph/ephrin transcripts. They found that binding of HuR protein (a member of the embryonic lethal abnormal vision family of RNA-binding proteins [39]) to these regions destabilized Eph/ephrin transcripts in tumor cell lines [40]. 

The interaction of Eph receptors and ephrins with other adhesion molecules such as E-cadherin could influence cell-cell attachment. Thereby, it is assumed that E-cadherin can influence the expression and cellular localization of Eph receptors and vice versa [41–43]. The modification of the cytoskeleton is another important prerequisite for enhanced cellular motility and invasion, respectively, and there exists evidence of involvement of Eph receptor/ephrin signaling. For instance, EphA3/ephrinA5 signaling induces growth cone collapse in retinal ganglion cells and cell rounding, blebbing, and detachment in EphA3-expressing human kidney epithelial cells and melanoma cell lines [44, 45]. In both studies it could be shown that Rho kinase is involved in the observed effects. This was further confirmed by Clifford and colleagues, who demonstrated that EphA3 receptor suppresses motility through regulation of Rho GTPases in rhabdomyosarcoma cell lines [46]. Moreover, Eph/ephrin signaling can influence cell survival as shown recently by Feng and colleagues. They demonstrated that overexpression of ephrinA2 in hepatocellular carcinoma cells leads to enhanced tumor cell survival and proved that this is caused by resistance to tumor necrosis factor-*α*-(TNF-*α*-) induced apoptosis [47]. In this regard, Holen et al. demonstrated in Jurkat TAg cells that signaling through ephrinA induced activation of Scr and Akt kinases, resulting in inhibition of antigen receptor-induced apoptosis [48]. Finally, it should be noted that some reports describe functionally relevant Eph receptor mutations in some tumor entities. For instance, mutations have been identified in EphA3 in melanoma and glioblastoma, and EphA3, EphA4, EphA7, and EphB6 in colorectal cancers [49, 50].

## 2. Angiogenesis

Angiogenesis is defined as growth of new blood vessels by sprouting from existing vessels [51, 52]. The lumen of blood vessels is faced by a single-layer squamous epithelium consisting of endothelial cells (ECs) which is separated from the circumjacent outer layers by the basal membrane. In small vessels (e.g., venules) ECs are enclosed by pericytes, in larger vessels by elastin fibres, smooth muscle cells and connective tissue. On one hand, ECs participate in the generation of blood vessels during embryonic development; on the other hand, they retain their ability to proliferation and migration in adult organisms, where they renew the inner wall of existing blood vessels and rebuild new vessels, for instance, in uterus mucosa during menstruation and wound healing. At the beginning of the formation of a new capillary ECs form lateral pseudopodia which develop to a hollow tube. This new capillary sprout expands until it meets another capillary sprout for fusion, resulting in blood flow. This process is regulated by different expression of surface molecules on arterial and venous capillaries. 

Angiogenesis is activated by signals from the target tissues. The reaction of the ECs spans four periods: secretion of proteases to cleave the basal membrane of the parental capillary, migration of ECs towards the signal, proliferation of ECs, and, finally, formation of tubes and differentiation of the ECs. Activating signals are soluble factors whose receptors are localized predominantly on ECs. The most important factor is vascular endothelial growth factor (VEGF) and its regulator hypoxia-inducing factor (HIF-1*α*), which stimulates transcription of the *VEGF* gene [51, 53]. Other important growth factors, like acidic and basic fibroblast growth factor (aFGF, bFGF), can also initiate angiogenesis, whereby they affect not only ECs but also other cell types [51]. Additional vascular ECs-specific growth factors involve four members of the angiopoetin family and at least one member of the ephrin family, whereby those factors have to operate highly coordinated to form functional vessels. Finally, factors not specific for ECs are required such as platelet-derived growth factor (PDGF) and tumor growth factor-*β* (TGF-*β*) [54]. Generally, it is assumed that VEGF functions as initiator of angiogenesis in development and adult organisms (with formation of immature vessels), followed by angiopoetin-1 and ephrinB2 function, necessary for maturation and stabilization of the vessel [54]. Moreover, angiogenesis is regulated not only by activating signals but also by inhibitors, for instance, thrombospondin-1, interferon-*α*, platelet factor-4, and angiostatin. To date, more than 20 inducers or inhibitors of angiogenesis have been identified [51]. 

### 2.1. Role of Eph Receptors and Ephrins in Angiogenesis

Concerning Eph/ephrin signaling in angiogenesis, the pair of EphB4/ephrinB2 seems to play a key role. They are assumed to define vascular borders due to their reciprocal distribution: ephrinB2 on arteries and EphB4 on veins already in early developmental stages [55–57]. The expression of ephrinB2 persists until late embryogenesis and adulthood, with distribution expanding from arterial ECs to surrounding smooth muscle cells and pericytes [54, 58]. Generally, interplay between ECs and perivascular supporting cells mediated by ephrinB2/Eph signaling is critical for vascular development as shown in several studies [30]. For instance, Foo and colleagues demonstrated that vascular smooth muscle cells require ephrinB2 for normal association with small-diameter blood vessels [59]. In this context, Oike et al. showed that unscheduled ubiquitous ephrinB2 expression in mice development leads to sudden death in embryonic stages due to defective recruitment of vascular smooth muscle cells to the ascending aorta [60]. Simultaneously, the authors suggest that bidirectional signaling is mandatory and that cell-to-cell repellent effects are important comparable to their role in the development of the central nervous system. In this regard, Füller et al. hypothesized that distinct propulsive and repulsive effector functions of endothelial ephrinB2 and EphB4 prevent intermingling of cells and mediate spatial position signals during angiogenesis and vessel assembly [61]. The importance of reverse signaling through ephrinB2 for vascular development is outlined by Adams et al. and analyzed in detail by Salvucci et al., who found that phosphorylation at the intracellular domain of ephrinB is dependent of Src kinases and is assumed to play a role in pericyte-to-ECs assembly into vascular structures [62, 63]. Additionally, migration and proliferation of ECs were analyzed by Steinle et al., who found that stimulation of EphB4 receptors with ephrinB2-Fc fragments leads to phosphorylation of Akt kinase and, furthermore, to increased proliferation and migration of the ECs. The authors show that this is mediated by the phosphatidylinositol 3-kinase/Akt/endothelial nitric-oxide synthase/protein kinase G/mitogen-activated protein kinase axis [64]. 

Beside EphB4/ephrinB2 other B-class Eph and ephrins play a role in vascularization and angiogenesis. In this regard, ephrinsB1, B3, and EphB2, B3, B4 are required for the regulation of the formation of the vascular network during cardiovascular development and for vascularization processes in the female reproductive system [65–67]. Furthermore, ephrinB1 is assumed to mediate ECs attachment on extracellular matrix by activation of integrins [68]. 

In the case of A-class Eph/ephrins, mainly EphA2 and ephrinA1 seem to be important for angiogenic processes. For instance ephrinA1 is expressed in vascular development during embryogenesis in murine endocardium, dorsal aorta and primary head veins and later in intersomitic vessels and the limb bud vasculature [69]. This implicates that ephrinA1 expression corresponds to regions of vasculogenesis and/or angiogenesis, and presumably enhances angiogenesis [55, 69]. Additional studies illuminated the role of involved pathways. Referring to this, the role of VEGF was analyzed by Cheng et al., who demonstrated that soluble EphA2-Fc receptors inhibited VEGF-induced survival, migration, sprouting of ECs and corneal angiogenesis [70]. The authors furthermore show that TNF-*α* induced ephrinA1 expression on ECs. This was found to be mediated by JNK and p38MAPK signaling pathways, leading to ECs migration and blood vessel assembly [71]. Another study showed that interaction of ephrinA1 with EphA2 induced activation of PI3 kinase and Rac1 GTPase leading to ECs aggregation and migration [72]. The role of EphA2/ephrinA1 in adult angiogenesis was further analyzed by different in vitro studies. For instance it was demonstrated that ephrinA1 enhanced assembly of human umbilical venous endothelial cells (HUVEC) in matrigel and that soluble EphA2-Fc receptors inhibited microvessel formation in a rat aortic ring assay [73, 74].

## 3. Tumor Angiogenesis

Angiogenesis can occur not only in physiological conditions but also in abnormal processes such as tumorigenesis. It is an early- to midstage event in many human cancers and a crucial step for the transition of a small, harmless cluster of mutated tumor cells into a large, malignant growth, capable of spreading to other organs throughout the body [75]. Without angiogenesis tumor size is restricted due to lack of nutrients, growth factors, and oxygen, resulting in counterbalance of dying and proliferating cells. Hypoxia in solid tumors occurs at a distance of ≥70 *μ*m from functional blood vessels and it is generally accepted that tumors do not exceed a volume of 1-2 mm^3^ without the induction of angiogenesis [51, 76]. Tumor angiogenesis starts with the appearance of proteins that promote neovascularization (angiogenesis). Such proteins are produced by tumor cells themselves or by infiltrating immune cells, such as macrophages [77]. Alternatively, angiogenic proteins can be mobilized by tumor cells from the nearby tissue. Once the process is initiated it cannot be controlled or even stopped by the malignant cells [75]. Instead, newly dividing ECs release different proteins that can stimulate the proliferation or motility of tumor cells, leading to support of metastasis. 

Generally, tumor cells produce two types of protein: one kind stimulates angiogenesis the other inhibits it, which lead to the hypothesis of an angiogenic switch in tumor angiogenesis [51, 76]. The most prominent angiogenic inducers are bFGF, aFGF, and VEGF with their corresponding receptors on ECs and among inhibitors are *α*-Interferon, platelet factor-4, and thrombospondin-1 [51]. FGF and other angiogenic factors can be sequestered in the extracellular matrix of many cell types, for instance ECs, and is believed to be released by proteolytic degradation of the extracellular matrix [51, 78]. For inhibitors alternative storage mechanisms are described: they are assumed to be stored as cryptic parts of larger molecules that are not per se inhibitors. Among them are a 29 kDa fragment of fibronectin [79], a 16 kDa fragment of prolactin [80, 81], angiostatin as fragment of plasminogen [82], a small fragment of platelet factor-4 [83], a propeptide of type 1 collagen [84], and a peptide fragment of endothelial growth factor [85]. The balance between angiogenic inducers and inhibitors determines whether the tumor can switch on angiogenesis, whereby tumor angiogenesis is preferentially induced by a loss or decrease in the production of inhibitors. Nevertheless, the underlying mechanisms are still poorly understood and dysregulation of transcription or the activation of different proteases are under discussion. 

An alternative way to facilitate tumor perfusion independent of tumor angiogenesis is the concept of vasculogenic mimicry [86, 87]. Thereby it is assumed that tumor cells re-express endothelial and mesenchymal markers, normally appearing on embryonic cells. This is accompanied by induction of vascular structures mimicking blood vessels and thus promoting tumor growth. For instance, metastatic melanoma cells are able to constitute channels filled with blood cells. This tubules exhibit a basal lamina but no ECs and the formation seems not to be dependent of bFGF, TGF-*β*, VEGF, PDGF, TNF-*α*, hypoxia, or integrins [87, 88]. In consequence, the formation of tubular networks on one hand results in better supply with nutrients and oxygen, on the other hand it can facilitate the invasion of tumor cells into the blood flow, thus, promoting metastasis [89]. Although the underlying mechanisms are not fully understood, the involvement of receptor tyrosine kinases, especially Eph receptors, is strongly suggested. In an in vitro study Hess and colleagues showed that transient knockout of EphA2 expression in aggressive uveal melanoma tumor cells resulted in inhibition of tubular network formation [88]. Further the authors found that phosphorylation of EphA2 by ephrinA1 leads to activation of downstream signaling kinases such as FAK and PI3 kinase and, furthermore, to the formation of vessel-like networks [90].

### 3.1. Role of Eph Receptors and Ephrins in Tumor Angiogenesis

The first reports concerning a direct connection between Eph receptor/ephrin signaling and tumor angiogenesis appeared approximately 10 years ago. Nikolova and colleagues investigated the B-class Eph receptors and ephrins and found a spatially, temporarily, and hormonally coordinated expression of EphB4 and ephrinB2 during normal mouse mammary morphogenesis. The receptor was predominantly localized in the myoepithelial cells surrounding the ducts and alveoli whereas ligand expression was limited to the luminal epithelial cells [91]. The disruption of the balanced expression lead to onset of carcinogenesis with loss of ligand expression and shift of receptor expression from myoepithelial cells surrounding the ducts to ECs with progressive malignancy [91]. The importance of EphB4/ephrinB2 in tumor angiogenesis and tumor growth was also demonstrated in recent work on mouse models. In this regard, Kimura and colleagues found that soluble ephrinB2-Fc molecules suppressed growth of head and neck squamous cell carcinoma xenografts by inducing maturation of vessels in the tumor [92]. Other studies investigating the effects of EphB4/ephrinB2 on tumor microvasculature, tumor growth, and survival of tumor cells indicated that EphB4 could act as a survival advantage in head and neck squamous cell carcinoma and in breast cancer, respectively [93, 94]. Class A molecules were analyzed by Ogawa et al. using two xenograft models from human breast cancer and Kaposi sarcoma. They found both ephrinA1 and EphA2 expressed in tumor cells and endothelial cells in these xenografts, and also in vasculature and tumor cells of surgically removed human cancers [95]. A further study revealed EphA2, in combination with VEGF, to be overexpressed in squamous cell carcinoma of oral tongue and, therefore, implicated in malignancy [96]. Today it is known that Eph receptors and ephrins are expressed in both tumor cells and tumor vasculature of many types of cancer, often at higher levels than in endogenous tissue [30]. Thereby, Eph receptor activation (forward signaling) is important as demonstrated by different studies using soluble receptors. Blocking EphA receptor signaling using soluble EphA2-Fc and EphA3-Fc receptors decreased tumor vascular density, tumor volume and cell proliferation in vivo, suggesting that the soluble receptors inhibited blood vessel recruitment by the tumor [74, 97, 98]. Furthermore, EphA2 kinase function in the tumor microenvironment seems necessary not only for tumor angiogenesis but also for metastatic progression [99, 100].

Nevertheless, reverse signaling through ephrins is another important factor in tumor angiogenesis. Expression of truncated, soluble EphB4 receptor in breast cancer cells in a mouse xenograft model (with ephrinB2 ligand primarily expressed in the vasculature) increased tumor angiogenesis, suggesting that soluble EphB4 promotes tumor growth by stimulating angiogenesis through ephrinB2 signaling [101]. Another study showed that EphB4 and ephrinB2 are expressed by ECs of human malignant brain tumors and overexpression of different EphB4 variants in blood vessels in tumor xenografts leads to the assumption that EphB4 acts as negative regulator of blood vessel branching and vascular network formation [102]. The involvement of additional Eph receptors in the switch of dormant tumors to the fast-growing angiogenic phenotype was analyzed recently by Almog and colleagues, who found increased EphA5 plasma levels in mice and, furthermore, that mRNA levels in tumor specimens of glioma patients correlated with disease stage. Hence, among other investigated molecules, EphA5 receptor possibly could represent a novel early cancer biomarker [103].

An important question remains unanswered, concerning the initiation of the altered Eph receptor/ephrin expression in tumor cells and tumor vasculature. Until now it is not fully understood which mechanisms lead to this dysregulation, but it is hypothesized that hypoxia could play a role in this context. For instance in a mouse skin flap model of hypoxia Vihanto and colleagues showed that hypoxia upregulates not only HIF-1*α* and VEGF but also EphB4, ephrinB2, EphA2 and ephrinA1 both on mRNA and protein levels up to 48 hours after induction of hypoxia [30, 104]. Furthermore, transcriptional profiles of umbilical cord blood and bone marrow-derived stem and progenitor cells showed that *EphA3* gene (among many other genes) is upregulated after hypoxia [105]. Another study, using HIF-2*α* knockdown mice showed that also HIF-2*α* interacts in hypoxia-induced tumor vascularization through activation of at least ephrinA1 [106]. In contrast, in neonatal rats exposed to chronic hypoxia, among others, expression of HIF-2*α* and ephrinA1 was downregulated [107]. However, it remains an important field and the identification of regulating mechanisms could provide novel targets for anti-angiogenic cancer therapies.

## 4. Therapeutical Interventions Targeting Eph Receptors and Ephrin Ligands

In contrast to many other therapeutic approaches, anti-angiogenic therapy does not aim to destroy tumor cells directly. Instead, it prevents tumor growth by its insufficient supply with nutrients and oxygen as a result of omitted blood vessel formation [75]. Numerous small molecule inhibitors and neutralizing antibodies targeting regulators of angiogenesis such as VEGF/VEGF receptors are recently under development and in clinical evaluation [108]. For instance, recently the Food and Drug Administration of the U.S.A. approved the anti-VEGF-A-neutralizing antibody Bevacizumab for treatment of stage III-IV colorectal cancer in combination with chemotherapy and for treatment of nonsquamous non-small cell lung cancers, as well as small molecule tyrosine kinase inhibitors for treatment of renal cell cancer (Sorafenib, Sunitinib) and hepatocellular carcinoma (Sorafenib) [109]. As Eph receptors and ephrins are also significantly involved in angiogenesis and tumor angiogenesis and, therewith, in tumor progression and metastasis, they represent important targets for cancer therapy [19, 30].

**Figure 3:**
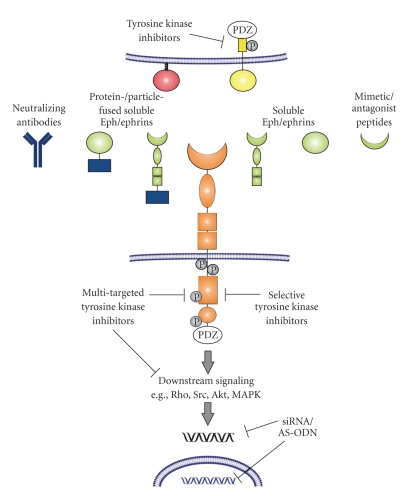
Potential target sites for Eph receptor/ephrin-associated antiangiogenic therapy. The illustrated strategies for intracellular inhibition of forward signaling via kinase inhibitors and gene silencing, respectively, also could be applied for reverse signaling. PDZ: Post synaptic density 95-Discs large-Zonula occludentes-1-protein, siRNA: small interfering RNA, AS-ODN: antisense oligodeoxynucleotides, Rho: Rho-GTPase Src: Src kinase, Akt: Akt kinase, MAPK: mitogen-activated protein kinase.

To date, there are different approaches to target Eph receptors and/or ephrins, either extracellularly by preventing receptor-ligand interactions or intracellularly through inhibition of tyrosine kinases or modification of gene transcription or translation (Figure 3). One of them is the application of monoclonal antibodies, which show high specificity and are already well established tools in tumor therapy. The first ones were directed against EphA2 and showed a significant inhibition of tumor growth in vitro [110, 111]. Furthermore, effective targeting and internalizing into antigen-positive tumors in different mouse xenograft models have been reported for EphA3 and EphB2 monoclonal antibodies [112, 113]. Although the specificity for a particular binding partner is probably limited, another approach with great potential represents blocking of the Eph receptor/ephrin signaling between tumor cells and ECs by the introduction of soluble Eph receptors. In this regard, it was demonstrated that soluble monomeric EphB4 receptor resulted in dramatically reduced tumor growth in mouse models [114, 115]. Furthermore, Scehnet and colleagues fused the extracellular domain of EphB4 with human serum albumin for blocking ephrinB2 which results in inhibited migration and invasion of Kaposi sarcoma cells in response to various growth factors [116]. In addition, the role of A-class Eph receptors was analyzed and inhibition of tumor angiogenesis and suppressed tumor growth in vivo was demonstrated for soluble EphA2-Fc and EphA3-Fc receptors [74, 97, 98]. Not only Eph receptors but also ephrins show therapeutic potency as truncated soluble forms. In this regard, soluble, monomeric ephrinA1 is a functional ligand for EphA2 in glioblastoma multiforme and modulates processes relevant to the progression of malignancy [10]. Beyond tumor pathology, soluble ephrinB2-Fc or EphB4-Fc chimeras, respectively, and soluble ephrinB2 were shown to reduce pathologic neovascularization in the retina [117, 118]. Moreover, a possible therapeutic strategy represents conjugation of ephrins to gold-coated silica nanoshells, which was used to selectively target prostate tumor cells [119]. An alternative strategy for targeting Eph receptor/ephrin signaling is the application of mimetic or antagonist peptides, which were generated so far for A-class as well as for B-class Eph receptors [120–123]. Finally, an alternative “extracellular” strategy is described by Yamaguchi and colleagues who investigated peptide-pulsed dendritic cell vaccines and found that immunization with dendritic cells pulsed with EphA2-derived peptides inhibited tumor growth in vivo in EphA2-positive murine colorectal adenocarcinomas [124].

Therapeutical strategies focusing on intracellular structures involve inhibitors, selective for a single or for multiple tyrosine kinases. In this regard, several 2,5-dimethylpyrrolyl benzoic acid derivatives have been generated as selective small molecule inhibitors for EphA4 receptors, as well as 2,4-bis-anilinopyrimidines for the inhibition of EphB4 receptors [125–127]. In addition, various *N*-substituted 3-amino-4-methylbenzamide based type II kinase inhibitors were analyzed concerning their potency to inhibit EphB2 receptor [128]. A well-characterized multiple-targeted tyrosine kinase inhibitor is dasatinib. It is a dual Src/Abl kinase inhibitor, whereby FAK, Crk-associated substrate, and EphA2 receptor are assumed as additional targets. The inhibitor shows potent anti-proliferative activity against hematologic malignancies [129] and has recently been approved for treatment of all stages of chronic myelogeneous leukemia [130]. Beneath its therapeutic effects in leukemias it was shown that dasatinib blocks migration and invasion of human melanoma cells without affecting proliferation and survival [130]. Furthermore, it was demonstrated that dasatinib blocks growth, migration and invasion of breast cancer cells [131], induced apoptosis and inhibited proliferation and invasion in different ovarian cancer cell lines [132]. Of importance, dasatinib also showed therapeutic potency to inhibit EphA2 in pancreatic cancer [133]. An additional conceivable approach for therapies directed against intracellular targets is the regulation of the gene expression using small interfering RNA or antisense oligodeoxynucleotides. In this regard, Kumar et al. demonstrated that knockdown of EphB4 expression leads to anti-tumoral effects in breast cancer in vitro and in vivo [93]. Furthermore, it was demonstrated that knockdown of EphA2 suppressed ephrinA1- and VEGF-induced endothelial cell migration and inhibited cell proliferation and induced apoptosis in human glioma cells [70, 134].

In part the pharmacological approaches against Eph receptor-/ephrin-mediated tumor angiogenesis discussed above also provide the possibility to develop strategies for imaging of tumor vascularization, for instance, by means of fluorescent- or radiolabeled-small molecule kinase inhibitors or peptide ligands. 

Overall, difficulties targeting Eph receptor/ephrin signaling in cancer therapy should be kept in mind. Heterogenous expression patterns of various Eph receptors/ephrins in tumor and normal tissue complicate the discrimination of malignant cells from nonmalignant cells [135]. Furthermore, the effects of Eph receptor-targeting agents on normal epithelial cells are insufficiently analyzed until today [136]. Another limitation in targeting Eph receptors represents the occasional opposing effects of one Eph receptor as tumor suppressor and tumor promoter [136]. In this regard, signaling of ephrinA1 and tumor cell-specific EphA2 suppresses processes like growth and migration, whereas interaction of ephrinA1 with ECs-specific EphA2 seems to stimulate these same effects [137]. Furthermore, the efficacy of EphA2 antibody-based therapy may depend on tumor type as no suppressive effect on tumor growth was observed in a colorectal tumor model [138], whereas mice harboring ErbB2 in mammary epithelium were sensitive to therapeutic inhibition of EphA2 [139]. When targeting the Eph kinase activity, it should be noted that inhibition is useful in tumors where kinase activity promotes tumorigenesis (melanoma) but may instead be ineffective or even detrimental for the treatment of other types of cancer where Eph receptor signaling suppresses tumorigenesis [136]. In addition, the binding promiscuity of Eph receptors and ephrin ligands as well as their capability to bidirectional signaling will further complicate targeting strategies and increase the potential for adverse side effects. Therapies designed to either activate or block an Eph receptor may also alter the signaling function of the ligand in adjacent cells [136, 140]. After all, possible interactions of Eph receptor/ephrin therapeutic agents with other agents should be considered. It is assumed, that the kinase inhibitor imatinib can counteract the anti-oncogenic effects of EphB4 agonists in breast cancer [136]. On the other hand, chemotherapeutic agents that target ErbB receptors may enhance the effects of EphB4-targeted therapies [136]. Despite and due to the mentioned limitations it is necessary to understand the complex functions of Eph receptors/ephrins in homeostasis and tumor progression to avoid undesirable side effects or unintentional exacerbation of disease functions [30]. In this regard, targeting Eph receptor/ephrin signaling to inhibit tumor angiogenesis and, therewith, tumor growth represents a promising approach in fighting cancer.

## 5. Conclusion

Eph receptors and their ligands, the ephrins, form a complex cellular communication system. Its complexity is based on the large number of different receptor and ligand molecules, their promiscuous binding properties, the ability to bidirectional signaling, formation of multimers, and crosstalk with other signaling pathways and molecules. An intricacy, we just begin to understand. Eph receptors and ephrins are involved in embryonic development, development of the nervous system, angiogenesis and also in tumorigenesis and tumor angiogenesis, respectively. They mediate cell-cell repellent effects, cell-cell and cell-matrix attachment, they influence cell survival and cytoskeleton dynamics, affecting cell motility, which could further result in tumor progression, invasion and metastasis. In the last decade Eph receptors and ephrin ligands were put in perspective to anti-tumoral and anti-angiogenic therapy. To date, many different therapeutic strategies targeting Eph receptors or ephrins are pursued and hopefully result in improvement of cancer treatment in the near future.
